# From Tradition to Bioactivity: Chemical and Biological Evaluation of a Reformulated Algerian Ancestral Soap Enriched With *Lavandula dentata* L. Essential Oil

**DOI:** 10.1002/cbdv.71753

**Published:** 2026-10-09

**Authors:** Manel Dirahoui, Farah Haddouchi, Rachida Amokrane‐aidat, Meknassia Debbal, Rayane Bougueroua

**Affiliations:** ^1^ Natural Products Laboratory, Department of Biology Abou Bekr Belkaid University Tlemcen Algeria; ^2^ Laboratory of Biomathematics, Biochemistry, Biophysics and Scientometry, Faculty of Natural and Life Sciences University of Bejaia Bejaia Algeria; ^3^ Laboratory of Chemistry, Physics, and Materials Biology, Department of Natural Sciences Higher Normal School of Technological Education of Skikda, Azzaba, Skikda Algeria

**Keywords:** antimicrobial activity, antioxidant activity, *Lavandula dentata* L., sustainability, traditional soap

## Abstract

The growing demand for sustainable and bioactive personal care products has renewed interest in traditional formulations. Saboun Dzair, a traditional Algerian olive oil‐based soap widely used in hammam practices, remains poorly investigated for its bioactive properties. *Lavandula dentata* L. essential oil (LDEO) was extracted by hydrodistillation from plants collected near Mostaganem, Algeria (yield: 0.10%). GC‐MS/MS analysis identified linalool (22.28%), linalyl acetate (14.42%), camphor, borneol, and 1,8‐cineole as major constituents. The extra virgin olive oil (EVOO) used for superfatting was dominated by oleic acid (60.97%). LDEO was incorporated at 5%, 8%, and 12% (w/w). DPPH antioxidant activity increased in a concentration‐dependent manner, from 9.09% inhibition in unenriched soap to 26.67% for 12% LDEO + 5% EVOO, an approximately 2.9‐fold enhancement. Antimicrobial assays against six microbial strains showed inhibition zones of 8–10 mm for unenriched soap and up to 24.96 mm for the optimized formulation (12% LDEO + 5% EVOO), with a MIC of 1.85 mg/mL against *Staphylococcus aureus*. These findings demonstrate that LDEO enrichment enhanced the antioxidant and antimicrobial properties of Saboun Dzair while supporting its potential as a sustainable, bioactive, and culturally relevant natural soap.

## Graphical abstract

Effects of thyroxine and nano‐melatonin supplementation on cryopreserved Simmental bull semen were evaluated. The combination of thyroxine (0.5 µg/dL) and nano‐melatonin (30 µg/dL) significantly improved post‐thaw sperm motility, membrane integrity, antioxidant status, and fertility while reducing oxidative stress and apoptosis. These findings demonstrate a synergistic cryoprotective effect during semen cryopreservation.

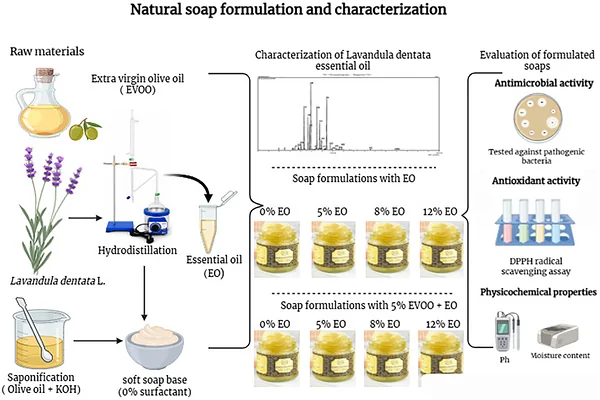

## 1 Introduction

The increasing global demand for eco‐friendly and bioactive personal care products has driven renewed interest in traditional cosmetic formulations. Consumers are increasingly seeking products that are not only effective and safe but also sustainable, culturally significant, and derived from natural sources. Among such sources, plant‐based ingredients, including essential oils and vegetable oils, have attracted considerable attention due to their antioxidant, antimicrobial, and skin‐beneficial effects [1, 2, 3]. Essential oils are particularly attractive for cosmetic applications because they can provide multiple functions within a formulation, serving not only as natural fragrance ingredients but also as active and potentially preservative agents with various skin‐beneficial properties [4, 5, 6, 7]. Despite this growing interest, the scientific validation of traditional cosmetic products remains limited, particularly in North African and Mediterranean contexts, where centuries‐old ethnobotanical knowledge has not yet been fully explored or integrated into modern formulations [8, 9]. This scientific valorization is particularly relevant for traditional products based on locally available botanical resources, as it can contribute simultaneously to the preservation of cultural heritage and the development of innovative natural cosmetic products.

*Lavandula dentata* L., a perennial aromatic shrub native to the Mediterranean basin, is widely recognized for its essential oil rich in biologically active compounds, including linalool, linalyl acetate, camphor, borneol, and 1,8‐cineole [10, 11]. These constituents are associated with antimicrobial, antioxidant, and anti‐inflammatory activities, making the species a promising candidate for enhancing the functional properties of cosmetic products [8, 12]. The chemical composition of *L. dentata* L. essential oil may, however, vary according to geographical origin, environmental conditions, and plant material, which can consequently influence its biological properties. Therefore, chemical characterization of the essential oil is an important prerequisite for establishing its potential application in a defined cosmetic formulation [4] While *L. dentata* L.is commercially cultivated in several Mediterranean countries, its integration into traditional Algerian cosmetics has been scarcely investigated [13]. In Algeria, Saboun Dzair is an emblematic traditional soap paste, historically formulated with virgin olive oil and widely used in hammam rituals for cleansing, exfoliation, and skin nourishment. Although its cultural and sensory value is well established, the bioactive potential of Saboun Dzair, particularly when enriched with plant‐derived compounds such as *L. dentata* L. essential oil, remains largely unexplored [13]. Moreover, modern consumers increasingly demand products that combine traditional knowledge with scientifically validated efficacy, highlighting a gap between ethnobotanical heritage and contemporary cosmetic science [14, 15]. Importantly, recent work on Algerian *L. dentata* L. essential oil has demonstrated its antioxidant, antibacterial, and antifungal potential, with reported MIC, MBC, and MFC values against several microorganisms [8]. These findings confirm the biological potential of Algerian *L. dentata* L. but also highlight the need to investigate its behavior and efficacy when incorporated into an actual cosmetic matrix rather than evaluated solely as an isolated essential oil [8]

In this context, the reformulation of traditional soaps with bioactive plant ingredients represents an interesting strategy for linking traditional knowledge with contemporary cosmetic research. However, the biological activity of an essential oil tested independently cannot necessarily be extrapolated to the finished product, since its activity may be influenced by the concentration used, the composition of the soap matrix, interactions with other ingredients, and the physicochemical environment of the formulation. Consequently, evaluation of the final formulated soap is essential to determine whether incorporation of the essential oil translates into measurable improvements in product functionality [6] The use of extra virgin olive oil (EVOO) as a superfatting agent provides an additional botanical component with potential functional relevance. Olive oil is characterized by a high proportion of oleic acid and contains minor compounds that may contribute to its antioxidant and skin‐conditioning properties. Thus, combining EVOO with *L. dentata* L. essential oil in a traditional soap matrix offers an opportunity to investigate the complementary contribution of two plant‐derived ingredients while maintaining the identity of the ancestral formulation.

This study aims to bridge this gap by valorizing Saboun Dzair through its enrichment with *L. dentata* L. essential oil and superfatting with extra virgin olive oil. The essential oil was chemically characterized by GC‐MS/MS, while the resulting soap formulations were evaluated for their physicochemical characteristics, antioxidant capacity, and antimicrobial activity. The antimicrobial assessment was complemented by quantitative determination of minimum inhibitory, bactericidal, and fungicidal concentrations to provide a more comprehensive evaluation of biological efficacy. By comparing the unenriched traditional soap with formulations containing increasing concentrations of *L. dentata L*. essential oil, this work specifically investigates whether enrichment can enhance the functional properties of Saboun Dzair without compromising its basic physicochemical characteristics. By integrating ancestral knowledge with modern analytical and biological evaluation techniques, this work contributes to the advancement of natural cosmetics while promoting the conservation and valorization of Algerian ethnobotanical heritage.

## 2 Results and Discussion

### 2.1 Chemical Composition of *Lavandula dentata* Essential Oil

The essential oil yield of *L. dentata* obtained in the present study was 0.10% (w/w). Similar variability in essential oil yield has been reported for this species across different regions of western Algeria. Bousmaha et al. (2006) investigated *L. dentata* collected from several locations and reported yields ranging from 0.40% to 0.79% in Enjajra (Tlemcen), 0.31%–0.59% in Souk El Khemis, 0.33%–0.47% in Sidi Driss, 0.25%–0.46% in Tafessout, 0.31%–1.04% in Honaine, 0.75%–0.98% in Ouled Echeikh Sid, 0.28%–0.55% in Souk El Tnine, and 0.24%–0.46% in Beni Saf [9].

Such variability in essential oil yield may be attributed to differences in bioclimatic conditions, geographical origin, soil properties, phenological stage at harvest, and environmental stress factors, which are known to significantly influence the biosynthesis and accumulation of volatile secondary metabolites in aromatic plants.

The physicochemical characterization of the essential oil revealed an optical rotation of −3.0° ± 0.02, suggesting the presence of optically active constituents that may contribute to its biological properties. The refractive index was 1.476 ± 0.013, indicating a satisfactory level of purity, while the density was 0.898 ± 0.004 g/cm^3^ at 20°C.

These values are in good agreement with those commonly reported for essential oils obtained from botanically related *Lavandula* species. In a study conducted in Brazil, Rocha et al. (2025) reported a density of 0.915 g/cm^3^ and a refractive index of 1.468 for *L. dentata* essential oil, which are comparable, although slightly higher and lower, respectively, than the values observed in the present study [10].

The chemical composition of the essential oil extracted from *L. dentata* and that of extra virgin olive oil was identified and quantified by gas chromatography–tandem mass spectrometry (GC–MS/MS) (Figure 1). A total of 100 compounds were identified in *L. dentata* essential oil, representing 99.98% of the total oil composition. Only compounds present at concentrations higher than 1% are reported in Table 1.

**FIGURE 1:**
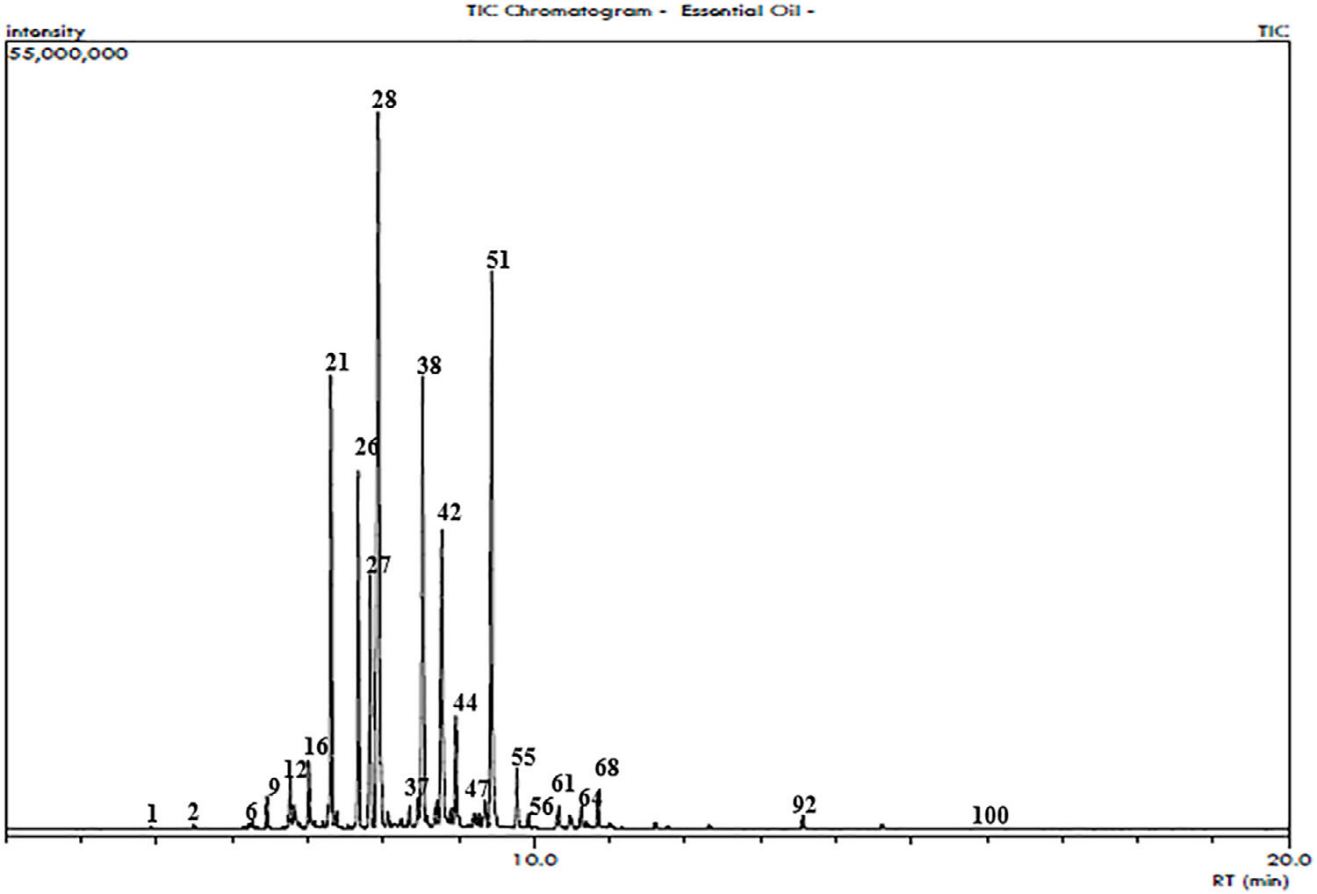
GC‐MS/MS chromatogram of *Lavandula dentata* essential oil.

**TABLE 1:** Chemical composition of *Lavandula dentata* L. essential oil: Major constituents (>1%) identified by GC–MS/MS.

Compound	CAS #	Molecular formula	RT^a^ (min)	RIexp^b^	RIlit^c^	SI (%)	Identification	% in EO
Acetic acid, hexyl ester	142‐92‐7	C_8_H_16_O_2_	7.014	1007	∼1012	98	RI, MS (NIST17)	1.54
Eucalyptol	470‐82‐6	C_10_H_18_O	7.309	1037	∼1029	97	RI, MS (NIST17)	8.86
*cis*‐Linalool oxide (furanoid)	5989‐33‐3	C_10_H_18_O_2_	7.671	1074	∼1069	97	RI, MS (NIST17)	7.25
*trans*‐Linalool oxide (furanoid)	34995‐77‐2	C_10_H_18_O_2_	7.825	1089	∼1086	96	RI, MS (NIST17)	5.03
Linalool	78‐70‐6	C_10_H_18_O	7.936	1101	∼1098	96	RI, MS (NIST17)	22.28
Camphor	464‐49‐3	C_10_H_16_O	8.522	1159	∼1143	98	RI, MS (NIST17)	10.98
Borneol	507‐70‐0	C_10_H_18_O	8.771	1184	∼1167	97	RI, MS (NIST17)	8.97
α‐Terpineol	98‐55‐5	C_10_H_18_O	8.961	1202	∼1188	95	RI, MS (NIST17)	2.80
Linalyl acetate	115‐95‐7	C_12_H_20_O_2_	9.437	1246	∼1257	98	RI, MS (NIST17)	14.87
Lavandulyl acetate	25905‐14‐0	C_12_H_20_O_2_	9.771	1277	∼1290	96	RI, MS (NIST17)	1.38

^a^ Retention time.

^b^ Experimental retention index, calculated using a homologous series of n‐alkanes (C_8–_C_20_) analyzed under identical chromatographic conditions on the Rxi‐5Sil MS column.

^c^ Literature retention index, compiled from Adams (2007) and Babushok et al. (2011) for comparable semi‐standard nonpolar (5%‐phenyl) stationary phases (tolerance ±20 RI units). SI: similarity index.

The essential oil was mainly characterized by a predominance of oxygenated monoterpenes. Monoterpene alcohols constituted the major chemical class, with linalool as the principal component (22.28%). Monoterpene esters, mainly linalyl acetate (14.42%), represented the second most abundant fraction, followed by bicyclic monoterpene ketones, dominated by camphor (10.98%). Bicyclic monoterpene alcohols, represented by borneol (8.97%), and monoterpene oxides, particularly eucalyptol (1,8‐cineole; 8.86%), were also present in significant proportions.

Linalool has been consistently reported as a major constituent of *L. dentata* essential oil from different geographical origins. In Morocco, Jilali et al. (2023) reported linalool contents ranging from 35% to 38%, depending on the extraction method, followed by linalyl acetate (∼28%) and camphor (∼12%) [11]. Similarly, El Abdali et al. (2022) identified linalool as the dominant compound (45.06%), with camphor and borneol as secondary constituents [8].

In contrast, a distinct chemotype was reported in Monte Verde, Brazil, where eucalyptol was the predominant compound (62.02%) and linalool accounted for only 7% of the total composition [10]. This marked variability highlights the influence of geographical origin, environmental conditions, and extraction techniques on the chemical profile of *L. dentata* essential oil, which is a key consideration for its industrial valorization.

(NIST17/W11N17MAIN1 spectral library match, acceptance threshold ≥85%). Compound identification was based on the combined agreement between RI concordance and mass spectral library matching; authentic reference standards were not coinjected.

### 2.2 Fatty Acid Composition of Extra Virgin Olive Oil

The fatty acid profile of the extra virgin olive oil (EVOO) used in this study showed a clear predominance of oleic acid (C18:1 n‐9), accounting for 60.97%, followed by linoleic acid (C18:2 n‐6; 15.63%), palmitic acid (C16:0; 15.20%), stearic acid (C18:0; 2.91%), and palmitoleic acid (C16:1 n‐7; 2.79%) (Table 2; Figure 2).

**TABLE 2:** Major constituents (>1%) of extra virgin olive oil identified by GC‐MS/MS analysis.

Compound	Fatty acid	CAS #	Molecular formula	RT^a^ (min)	SI^b^ (%)	% in EVOO
Hexadecanoic acid, methyl ester	Palmitic acid (C16:0)	112‐39‐0	C_17_H_34_O_2_	17.412	86	15.20
11‐Hexadecenoic acid, methyl ester	Palmitoleic acid (C16:1 n‐5)	55000‐42‐5	C_17_H_32_O_2_	17.597	94	2.79
Methyl stearate	Stearic acid (C18:0)	112‐61‐8	C_19_H_38_O_2_	19.171	96	2.91
9‐Octadecenoic acid (Z)‐, methyl ester	Oleic acid (C18:1 n‐9)	2462‐84‐2	C_19_H_36_O_2_	19.435	93	55.30
9‐Octadecenoic acid (Z)‐, methyl ester^*^	Oleic acid isomer (C18:1)	112‐62‐9	C_19_H_36_O_2_	19.483	96	5.67
9,12‐Octadecadienoic acid (Z,Z)‐, methyl ester	Linoleic acid (C18:2 n‐6)	112‐63‐0	C_18_H_32_O_2_	19.935	96	15.63

^a^ Retention time.

^b^ Similarity index (spectral library match, NIST17).

* Minor C18:1 isomer peak, tentatively assigned; exact identity unresolved by MS library matching alone.

**FIGURE 2:**
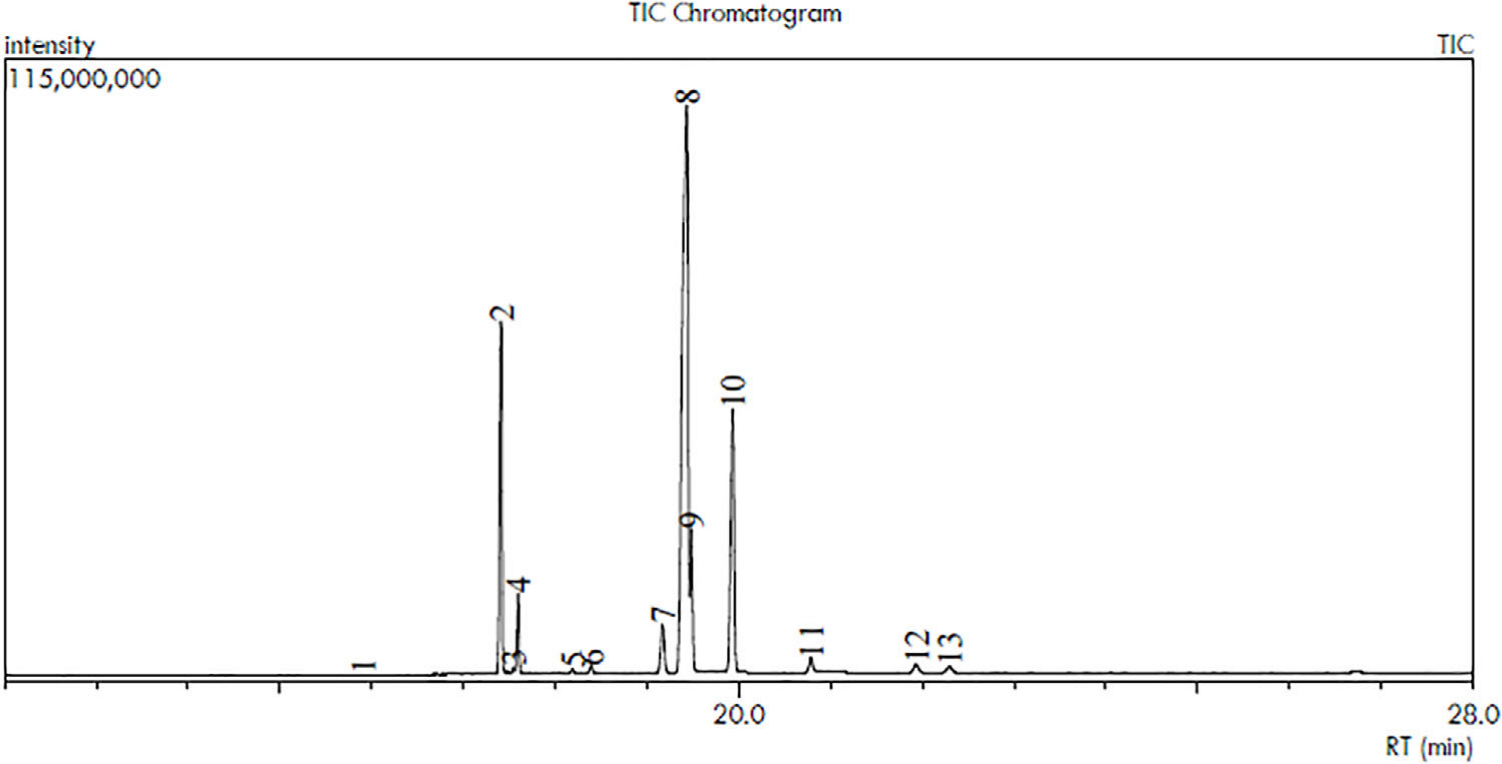
GC‐MS/MS chromatogram of extra virgin olive oil.

These values are consistent with those reported for high‐quality EVOO [12] and fall within the limits established by the International Olive Council. Although the oleic acid content was slightly lower than that reported in previous studies (68%–71%), it remains well within the IOC acceptable range (55.00%–83.00%). The relatively higher linoleic acid content may be attributed to varietal characteristics, climatic conditions, or a more advanced ripening stage at harvest.

Overall, the fatty acid composition confirms the suitability of the EVOO used in this study as a high‐quality lipid matrix for formulation and industrial applications.

### 2.3 Physicochemical Properties of Olive Oil Soft Soap Enriched With *L. dentata* Essential Oil

The physicochemical analysis of the prepared soaps is summarized in Figure 3 (numerical values are provided in Table S1). Moisture content ranged from 24.78% to 25.94%, with the control formulation exhibiting the highest value (25.86%), well below the maximum limit of 60% defined by SNI 06‐4085‐1996 [13]. No significant differences were observed among the formulations, indicating that neither *L. dentata* essential oil nor the addition of extra virgin olive oil significantly affected hydration. Maintaining adequate moisture is critical to ensure product stability, microbiological safety, and textural integrity during storage [14, 15].

**FIGURE 3:**
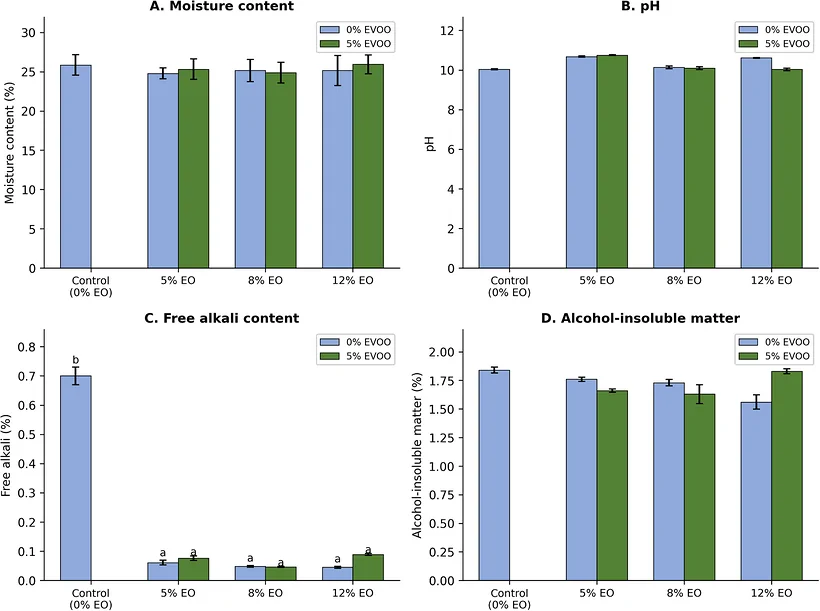
Physicochemical properties of olive oil soft soap formulated with varying levels of *L. dentata* essential oil (mean ± SD, *n* = 3; one‐way ANOVA, Tukey's posthoc test; bars sharing a letter are not significantly different, *p* < 0.05).

The pH values were between 10.03 and 10.75, reflecting the alkaline nature of soft soaps prepared with potassium hydroxide and the fatty acid profile of olive oil [16]. Although slightly higher than the upper limit suggested by SNI 2588:2017, these values are typical for traditional soft soaps and do not pose a risk for normal skin use.

Free alkali content was significantly reduced in all EO‐enriched formulations (0.045%–0.089%) compared to the control (0.70%), remaining below the 0.1% limit defined by EAS 186 (“Toilet soap—Specification”). This reduction is attributed to the 5% superfatting step, where excess fatty acids neutralize residual alkali, improving skin compatibility and moisturizing properties [17].

Alcohol‐insoluble matter ranged from 1.56% to 1.84%, below the 2% limit established by EAS 186. The high solubility in ethanol (up to 98.16%) indicates a low proportion of unreacted or unsaponified residues [18]. Overall, these results confirm that incorporation of *L. dentata* essential oil and the superfatting process enhance soap safety and quality without adversely affecting moisture, pH, or texture.

### 2.4 Antioxidant Activity

The antioxidant activity of *L. dentata* essential oil (EO), extra virgin olive oil (EVOO), and the formulated soaps was evaluated using the DPPH radical‐scavenging assay (Figure 4; numerical values are provided in Table S2). The EO showed moderate activity (IC_50_ = 8.72 ± 0.03 mg/mL), lower than the synthetic antioxidant BHT (0.0146 ± 0.0008 mg/mL) but higher than previously reported values for *L. dentata* from Morocco (12.95 mg/mL) [4]. Tunisia (14.03 mg/mL) [15], and *Lavandula angustifolia* from Italy (26.26 mg/mL) [19]. These differences likely reflect chemotypic variability and environmental factors influencing EO composition [20, 21].

**FIGURE 4:**
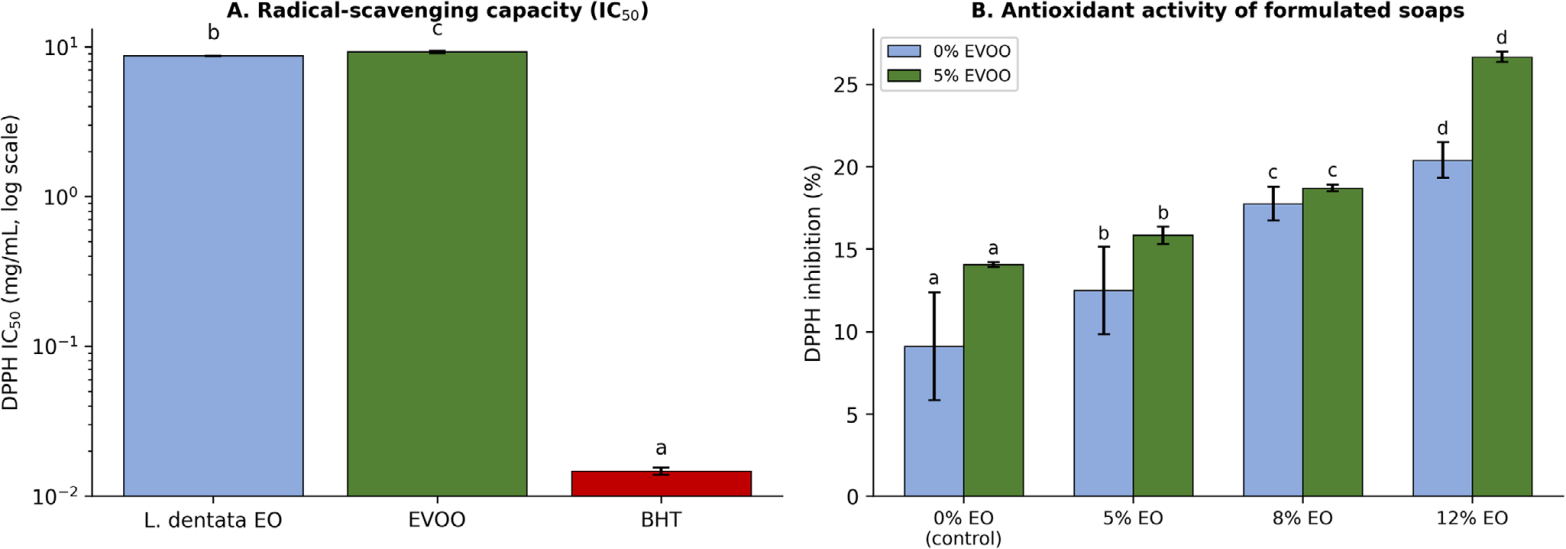
Antioxidant activity (DPPH assay) of *L. dentata* essential oil, extra virgin olive oil, BHT, and formulated soaps (mean ± SD, *n* = 3; one‐way ANOVA, Tukey's posthoc test; bars sharing a letter are not significantly different, *p* < 0.05).

The moderate antioxidant potential of *L. dentata* EO can be attributed to its chemical profile, dominated by oxygenated monoterpenes such as linalool, linalyl acetate, borneol, and eucalyptol, which are known to donate hydrogen atoms and stabilize reactive oxygen species [22, 23]. Minor compounds may contribute synergistically to the overall radical‐scavenging activity.

EVOO also exhibited strong antioxidant activity (IC_50_ = 9.25 ± 0.19 mg/mL), consistent with its high content of phenolic compounds and vitamins A and E [24, 25, 26]. Its activity is mainly attributed to phenolic–lipid interactions, while fatty acids such as palmitic and oleic acids have limited radical‐scavenging activity [27].

The incorporation of *L. dentata* EO into soap formulations significantly enhanced antioxidant activity in a concentration‐dependent manner. Soaps without EVOO showed DPPH inhibition from 9.09% (0% EO) to 20.40% (12% EO), while soaps with 5% EVOO reached 26.67% (12% EO), demonstrating a synergistic effect between EO monoterpenes and olive oil polyphenols [28, 29]. However, the antioxidant activity of the formulated soaps remained lower than that of pure EVOO, likely due to matrix effects of saponified fatty acids, which can partially immobilize phenolic and terpenoid compounds, limiting their availability to scavenge radicals [14, 30].

Overall, these findings indicate that *L. dentata* EO can act as a natural antioxidant in cosmetic formulations, and its combination with EVOO further improves the radical‐scavenging potential of soft soaps.

### 2.5 Antibacterial and Antifungal Activities by Disk Diffusion Assay

The antimicrobial activities of *L. dentata* essential oil (EO), extra virgin olive oil (EVOO), and the formulated soaps were evaluated against Gram‐positive and Gram‐negative bacteria and *Candida albicans* using the disk‐diffusion assay (Table 3). Traditional Dzair soap exhibited the weakest antimicrobial activity, with inhibition zones ranging from 8.35 to 10.00 mm across the tested microorganisms. The relatively limited antimicrobial activity of the unenriched soap may be related to its primary cleansing function, whereas incorporation of bioactive botanical ingredients can improve the functional properties of soap formulations [31, 32]. The incorporation of *L. dentata* EO markedly enhanced the antimicrobial activity of the soap formulations in a concentration‐dependent manner. In formulations without EVOO, increasing the EO concentration from 5% to 12% progressively increased the inhibition zones against all tested microorganisms. At 12% EO, inhibition zones reached 19.50, 18.80, 20.40, 14.65, 13.55, and 16.50 mm against *Escherichia coli*, *Staphylococcus aureus*, *Staphylococcus epidermidis*, *Klebsiella pneumoniae*, *Pseudomonas aeruginosa*, and *C. albicans*, respectively. A similar trend was observed in formulations containing 5% EVOO, with the 12% EO + 5% EVOO formulation producing inhibition zones ranging from 16.00 to 23.55 mm.

**TABLE 3:** Antimicrobial activity of *L. dentata* essential oil and formulated soap samples against the tested microorganisms, expressed as inhibition‐zone diameters (mm).

Samples	*Escherichia coli*	*Staphylococcus aureus*	*Staphylococcus epidermidis*	*Klebseilla pneumoneae*	*Pseudomonas aerugenosa*	*Candida albicans*
L. dentata EO	40.00 ± 1.41^A^	29.00 ± 1.41 ^A^	26.88 ± 1.58 ^A^	46.00 ± 1.41^A^	19.40 ± 0.56^A^	22.25 ± 1.06 ^A^
Virgin olive oil	11.5 ± 0.70^EF^	11.65 ± 0.49 ^EF^	10.6 ± 0.56 ^FG^	10.15 ± 0.35^DE^	10.60 ± 0.56^DE^	9.6 ± 0.86^E^
Soap	9.00 ± 1.41^F^	10.00 ± 1.41 ^F^	9. 85 ± 0.21^G^	9.10 ± 0.56^E^	9.07 ± 0.81 ^E^	8.35 ± 0.91^E^
Soap with 0% extra virgin olive						
5% *L. dentata* EO	13.65 ± 0.49 ^DEF^	12.25 ± 0.35 ^EF^	13.50 ± 0.70 ^EF^	10.00 ± 1.41^DE^	11.00 ± 1.41^CDE^	11 ± 1.41^DE^
8% *L. dentata* EO	16.55 ± 1.20 ^BCD^	14.60 ± 0.56 ^DE^	16.85 ± 0.21^D^	12.00 ± 1.41^BCDE^	12.85 ± 1.20 ^BCDE^	13.85 ± 1.20^BCD^
12% *L. dentata* EO	19.50 ± 1.13 ^BC^	18.80 ± 0.28 ^BC^	20.40 ± 0.56^BC^	14.65 ± 0.91^BC^	13.55 ± 1.06^BCD^	16.5 ± 0.98^B^
Soap with 5% extra virgin olive						
5% *L. dentata* EO	14.75 ± 1.06 ^CDE^	14.1 ± 0.84 ^DE^	14.80 ± 1.13^DE^	11.00 ± 1.41^CDE^	12.95 ± 1.34 ^BCDE^	12.5 ± 0.71^CDE^
8% *L. dentata* EO	16.55 ± 1.48 ^BCD^	15.95 ± 0.35^CD^	17.75 ± 0.35^CD^	13.75 ± 0.35^BCD^	14.75 ± 1.06^BC^	16.25 ± 1.06^BC^
12% *L. dentata* EO	20.9 ± 1.69 ^B^	20.60 ± 0.56^B^	23.55 ± 0.77^B^	16.00 ± 1.41^B^	16.36 ± 0.54 ^AB^	17.93 1.15B

Data are expressed as mean values ± standard deviation. A–G: means in the same column with different letters are significantly different (*P* < 0.05).

The pure *L. dentata* EO exhibited considerably larger inhibition zones than the formulated soaps, ranging from 19.40 to 46.00 mm, confirming its strong intrinsic antimicrobial activity. This result is consistent with previous studies reporting antibacterial and antifungal activities of *Lavandula* essential oils [33, 34, 35, 36]. The lower inhibition zones observed for the formulated soaps compared with the pure EO may be related to the physicochemical characteristics of the soap matrix, which can affect the release and diffusion of volatile constituents through the agar medium [34, 35, 36]. However, because essential oils are complex mixtures, the observed antimicrobial activity is more appropriately attributed to the combined effects of several constituents rather than to a single major compound.

The formulations containing both EO and EVOO generally exhibited higher inhibition zones than the corresponding EO‐only formulations. For example, the 12% EO + 5% EVOO formulation produced inhibition zones of 20.90, 20.60, 23.55, 16.00, 16.36, and 17.93 mm against *E. coli*, *S. aureus*, *S. epidermidis*, *K. pneumoniae*, *P. aeruginosa*, and *C. albicans*, respectively. The enhanced activity observed after the addition of EVOO may reflect a complementary contribution of the two botanical ingredients. However, a specific synergistic interaction cannot be established solely from inhibition‐zone measurements and would require dedicated combination assays. The antimicrobial potential of olive oil has previously been reported [32]. Importantly, the antimicrobial evaluation was not limited to the qualitative disk‐diffusion assay. Quantitative determination of the minimum inhibitory concentration (MIC) and minimum bactericidal/fungicidal concentrations (MBC/MFC) was also performed for *L. dentata* EO. The EO showed MIC values ranging from 1.85 to 12.75 mg/mL, with the lowest values observed against *S. aureus* (1.85 mg/mL) and *S. epidermidis* (2.25 mg/mL). The corresponding MBC/MIC ratios were approximately 2.16 and 2.40, respectively, indicating a bactericidal effect. For *C. albicans*, the MFC/MIC ratio was 2.60, supporting a fungicidal effect. Gram‐negative bacteria exhibited comparatively higher MIC and MBC values, which may be related to the additional permeability barrier provided by their outer membrane.

Overall, the disk‐diffusion and quantitative MIC/MBC/MFC results consistently demonstrate that incorporation of *L. dentata* EO enhances the antimicrobial performance of traditional Dzair soap. The 12% EO + 5% EVOO formulation showed the highest antimicrobial activity among the formulated soaps, while the pure EO exhibited the strongest activity overall. These findings support the potential of *L. dentata* EO as a bioactive botanical ingredient for the reformulation of traditional Algerian soap.

### 2.6 Minimum Inhibitory and Bactericidal/Fungicidal Concentrations

The quantitative antimicrobial activity of *L. dentata* essential oil was further assessed by determining the minimum inhibitory concentration (MIC) and minimum bactericidal/fungicidal concentration (MBC/MFC) against the tested microorganisms (Table 4). The MIC values ranged from 1.85 to 12.75 mg/mL, indicating marked differences in susceptibility among the tested microorganisms. The lowest MIC was observed for *S. aureus* (1.85 mg/mL), followed by *S. epidermidis* (2.25 mg/mL), whereas the highest MIC was recorded for *P. aeruginosa* (12.75 mg/mL). *E. coli*, *K. pneumoniae*, and *C. albicans* showed intermediate MIC values of 3.50, 7.25, and 5.75 mg/mL, respectively.

**TABLE 4:** Minimum inhibitory, bactericidal, and fungicidal concentrations of *Lavandula dentata* L. essential oil against the tested microorganisms.

Microorganism	MIC (mg/mL)	MBC/MFC (mg/mL)	MBC/MIC or MFC/MIC
*Escherichia coli*	3.50	15.70	4.48
*Staphylococcus aureus*	1.85	4.00	2.16
*Staphylococcus epidermidis*	2.25	5.40	2.40
*Klebsiella pneumoniae*	7.25	32.85	4.53
*Pseudomonas aeruginosa*	12.75	55.00	4.31
*Candida albicans*	5.75	15.00	2.60

Abbreviations: MBC, minimum bactericidal concentration; MFC, minimum fungicidal concentration.

The MBC/MFC values followed a similar pattern. The lowest MBC was observed against *S. aureus* (4.00 mg/mL), followed by *S. epidermidis* (5.40 mg/mL), whereas *P. aeruginosa* required the highest concentration (55.00 mg/mL). The relatively low MIC and MBC values obtained for the two Gram‐positive staphylococci indicate greater susceptibility to the EO compared with the tested Gram‐negative bacteria. This difference may be related, at least in part, to structural differences in the bacterial envelope. Gram‐negative bacteria possess an additional outer membrane that can restrict the penetration of hydrophobic essential‐oil constituents, whereas Gram‐positive bacteria lack this additional permeability barrier [37].

The MBC/MIC ratios provided further information regarding the nature of the antimicrobial effect. Ratios of 2.16 for *S. aureus* and 2.40 for *S. epidermidis* indicate a bactericidal effect according to the commonly applied MBC/MIC criterion. In contrast, the ratios obtained for *E. coli* (4.48), *K. pneumoniae* (4.53), and *P. aeruginosa* (4.31) were slightly above the conventional threshold of 4, suggesting a predominantly inhibitory effect under the conditions tested. For *C. albicans*, the MFC/MIC ratio was 2.60, indicating a fungicidal effect. The pronounced susceptibility of the two staphylococcal species and the fungicidal effect observed against *C. albicans* further support the broad antimicrobial potential of *L. dentata* EO.

The comparatively high MIC and MBC values observed for *P. aeruginosa* are consistent with its recognized intrinsic resistance to many antimicrobial agents and may be associated with the low permeability of its outer membrane and other resistance mechanisms. *K. pneumoniae* also exhibited relatively high MIC and MBC values, whereas *E. coli* showed intermediate susceptibility. Similar differences in susceptibility among microorganisms have been reported for essential oils and may reflect differences in cell‐envelope structure and permeability [37].

The antimicrobial activity observed in the present study may be associated with the chemical composition of *L. dentata* EO, which was dominated by oxygenated monoterpenes, particularly linalool, linalyl acetate, camphor, borneol, and eucalyptol. Previous studies have reported antimicrobial activity for *Lavandula* essential oils and have linked their biological effects to their complex mixture of volatile constituents [35, 36]. The antimicrobial action of these compounds may involve interactions with microbial cell membranes, resulting in altered membrane permeability and disruption of cellular homeostasis. Nevertheless, because essential oils contain numerous constituents that may act individually or in combination, the contribution of any single compound cannot be established from the present data alone.

The MIC, MBC, and MFC results complement the disk‐diffusion findings obtained for the soap formulations. In particular, the superior inhibition zones observed for formulations containing both EO and EVOO suggest that the combination of the two botanical ingredients can improve the antimicrobial performance of the soap matrix [38]. However, the present data do not allow a definitive synergistic interaction to be established, as this would require a dedicated combination assay such as a checkerboard analysis. Nevertheless, the enhanced activity observed for the 12% EO + 5% EVOO formulation, together with the quantitative antimicrobial activity demonstrated for the pure EO, highlights the potential of this formulation as a functional natural soap [38, 39].

Overall, the combined disk‐diffusion and MIC/MBC/MFC results demonstrate that enrichment of traditional Dzair soap with *L. dentata* EO enhances its antimicrobial performance. The 12% EO + 5% EVOO formulation showed the highest antimicrobial activity among the formulated soaps, whereas the pure EO exhibited the strongest quantitative antimicrobial activity. These findings support the potential of the optimized formulation as a natural and culturally relevant personal‐care product with enhanced antibacterial and antifungal properties [39, 40].

## 3 Conclusions

The essential oil of *L. dentata* collected from the Aïn Sidi Cherif region is rich in oxygenated monoterpenes, conferring strong antioxidant and antimicrobial activities. Incorporation of this essential oil into traditional olive oil‐based soaps significantly enhanced their biological properties, with a notable synergistic effect between olive oil and the monoterpenes. Optimized formulations, particularly those containing 12 % essential oil and 5 % olive oil, provided a balanced combination of physicochemical stability and antimicrobial efficacy.

These findings highlight the potential of *L. dentata* as a natural ingredient for the development of functional soaps for cosmetic and hygienic applications. Further studies on long‐term stability and in vivo efficacy could support industrial valorization of this local resource.

## 4 Experimental Section

### 4.1 Plant Material

Fresh leaves of *L. dentata* were collected early in the morning at the end of the growth cycle (April 2025) from a natural population in the Ain Sidi Cherif region, near Mostaganem, northwestern Algeria (35.8361°N, 0.1284°E), at an altitude of approximately 120 m and 15 km from the Mediterranean Sea. The plant material was identified by Dr. Benzid Amel, ethnobotanist researcher at the University of Sciences and Technology Houari Boumediene (USTHB), Algeria, and taxonomically authenticated by the Department of Plant Conservation, Botanical Garden of El Hamma (Jardin d'Essai du Hamma), Algiers, Algeria. A voucher specimen was deposited at this institution; the corresponding accession number is currently pending confirmation from the herbarium and will be communicated to the Editorial Office as soon as it becomes available. Leaves were carefully washed with distilled water to remove debris, and then air‐dried in a dark, ventilated environment to preserve phytochemical integrity.

### 4.2 Extraction of Essential Oil

The essential oil (EO) was extracted from 100 g of dried leaves using hydrodistillation in a Clevenger‐type apparatus for 3 h, following the ISO 6571:2018 standard (ISO, 2018). The EO yield (%) was calculated as the volume of oil obtained per 100 g of dried plant material. The extracted EO was stored in amber glass vials at 4°C until further chemical and biological analyses.

### 4.3 Physicochemical Characterization of Essential Oil

The EO was characterized for physicochemical parameters according to ISO standards (ISO 3515:2004, ISO 6320, ISO 279:1998). Parameters evaluated included density, refractive index, optical rotation, and acidity. These analyses ensured the quality, reproducibility, and suitability of the EO for subsequent biological assays.

### 4.4 GC–MS/MS Analysis of the Essential Oil

The essential oil of *L. dentata* L. was analyzed using a Shimadzu GCMS‐TQ 8040 NX system equipped with an Rxi‐5Sil MS capillary column (cross‐bond 5% diphenyl/95% dimethylpolysiloxane; 30.0 m × 0.25 mm i.d., film thickness 0.25 µm). Analyses were carried out in split injection mode at 250°C with a split ratio of 60:1, and 2 µL of EO was injected. Helium was used as the carrier gas at a constant flow rate of 1.20 mL/min, corresponding to a linear velocity of 39.5 cm/s. The oven temperature program was: 40°C (held for 3 min), ramped at 25°C/min to 125°C, then ramped at 10°C/min to 300°C (held for 15 min), with a total run time of approximately 39 min.

Mass spectrometry conditions included an ion source temperature of 200°C, scan speed of 2500, and a mass scan range of *m*/*z* 30–500. Retention indices (RI) were calculated using a series of n‐alkane standards (C_8–_C_20_) under identical chromatographic conditions.Compound identification was performed using electron‐ionization (EI) mass spectra acquired in Q3 full‐scan mode (*m*/*z* 30–500, scan speed 2500), with the collision‐induced dissociation (CID) gas turned off; therefore, tandem mass spectrometry (MS/MS) fragmentation was not used for structural confirmation. The acquired mass spectra were compared with those in the NIST17 and Wiley (W11N17MAIN1) spectral libraries, and a similarity index (SI) of ≥85% was used as the acceptance threshold for reliable identification (66 of 100 detected peaks, representing 97.5% of total peak area; SI range 85%–98%). Linear retention indices (RI) were calculated according to the van den Dool and Kratz method using a homologous series of n‐alkanes (C8–C20) analyzed under identical chromatographic conditions on the same Rxi‐5Sil MS column. Experimental RI values (RIexp) were compared with literature RI values (RIlit) reported for comparable semi‐standard nonpolar stationary phases (5% phenyl), and agreement within ±20 RI units was considered acceptable. Authentic reference standards were not coinjected; consequently, compound assignments were based on the combined agreement between EI mass‐spectral library matching and experimental‐to‐literature RI concordance. The use of the triple‐quadrupole GC–MS/MS instrument in full‐scan EI mode reflects the instrumental configuration available at the analytical facility and does not imply that tandem‐MS fragmentation was used for compound confirmation.

### 4.5 Natural Soap Formulation and Characterization

Traditional Algerian soap (*Saboun Dzair*) was prepared from virgin olive oil using a hot saponification process with a 30% potassium hydroxide (KOH) solution. The KOH solution was gradually added to 100 g of olive oil under continuous stirring using a stick blender until a thick paste was obtained. The paste was heated at approximately 80°C for 3 h, with occasional stirring, until saponification was complete.

After saponification, the paste was divided into two portions: one without superfatting (0%), and the other enriched with 5% extra virgin olive oil (5% superfatting). *L. dentata* essential oil was incorporated at concentrations of 5%, 8%, and 12% (w/w) under continuous stirring. The soaps were cured at room temperature for 2 weeks before analysis.

The physicochemical parameters evaluated to assess soap quality included moisture content, pH, free alkali content, and alcohol‐insoluble matter, following ISO standards (ISO 672:1978, ISO 4316:1977, ISO 456:1973, ISO 673:1981) (San Miguel & Grice, 2015). Visual appearance and odor were also noted qualitatively.

### 4.6 Antioxidant Activity Assessment

The antioxidant activity of virgin olive oil, *L. dentata* EO, and formulated soaps was determined using the DPPH radical scavenging assay.

For essential oils and olive oil, the assay was conducted according to Atoui et al. (2005) with ethanol substituted for methanol [41]. The method is based on the ability of antioxidant compounds to reduce the DPPH radical, resulting in a decrease in absorbance at 517 nm. Greater absorbance reduction indicates higher antioxidant capacity. For essential oils and olive oil, 50 µL of each sample, at final concentrations of 5, 10, 15, 20, and 25 µL/mL, was added to 1950 µL of an ethanolic DPPH solution (6.34 × 10^−^^5^ M). A negative control (50 µL ethanol + 1950 µL DPPH solution) and a corresponding blank were prepared for each concentration. Mixtures were incubated in the dark at room temperature for 30 min, and absorbance was measured at 517 nm. Butylated hydroxytoluene (BHT) was used as the positive control (0.00156–0.025 mg/mL). Percentage inhibition (PI) was calculated as PI = [(A_control − A_sample)/A_control] × 100, and IC_50_ values were determined from dose‐response curves (*n* = 3). For the formulated soaps, the DPPH assay followed the protocol of Prieto Vidal et al. (2018) [42], with results expressed as percentage inhibition.

### 4.7 Antimicrobial Activity Assessment

The antimicrobial activity of *L. dentata* EO and formulated soaps was evaluated using the disc diffusion method [43]. Against six microbial strains: *S. aureus* (ATCC 6538), *S. epidermidis* (ATCC 12228), *E. coli* (ATCC 8739), *P. aeruginosa* (ATCC 27853), *K. pneumoniae* (ATCC 4352), *C. albicans* (ATCC 10231). Sterile discs were impregnated with 20 µL of EO; DMSO served as the negative control. Bacterial suspensions were standardized to 10^8^ CFU/mL (OD 0.08–0.13 at 625 nm) and spread on Mueller–Hinton agar plates using a sterile swab. Discs containing EO, soap, or antibiotics were aseptically placed on the agar surface. Plates were incubated at 37°C for 24 h, and zones of inhibition (mm) were measured. All experiments were performed in triplicate.

### 4.8 Statistical Analysis

Data were expressed as mean ± standard deviation (SD). Differences between groups were analyzed using one‐way ANOVA, followed by Tukey's post‐hoc test. Statistical significance was considered at *p* < 0.05. Analyses were performed using JMP Pro Version 14.0 (SAS, USA).

## Author Contributions

**Manel Dirahoui** performed the experimental study and wrote original draft. **Rachida Amokrane‐Aidat** provided resources and performed the experimental study. **Farah Hadouchi** and **Meknassia Debbal** conceptualized this study, interpreted the experimental data, and reviewed original draft. **Rayane Bougueroua** contributed to the interpretation of GC–MS data and the organization and preparation of the manuscript.

## Funding

This research received no specific grant from any funding agency in the public, commercial, or not‐for‐profit sectors.

## Conflicts of Interest

The authors declare no conflicts of interest.

## Supporting information




**Supporting File 1**: cbdv71753‐sup‐0001‐SuppMat.pdf

## Data Availability

The data supporting the findings of this study are available within the article and its Supporting Information.
