# Supplementary material for: From Tradition to Bioactivity: Chemical and Biological Evaluation of a Reformulated Algerian Ancestral Soap Enriched With Lavandula dentata L. Essential Oil
Source: Chem Biodivers. 2026 Oct 9;23(10):e71753. doi: 10.1002/cbdv.71753 (PMC13653130; doi:10.1002/cbdv.71753)
Supplement: Supplementary file 1 [file CBDV-23-e71753-s001.pdf]

Table of Contents

Table S1. Physicochemical properties of olive oil soft soap formulated with varying levels of *Lavandula dentata* essential oil

Table S2. Antioxidant activity (DPPH assay) of *Lavandula dentata* essential oil, extra virgin olive oil, BHT, and formulated soap samples

Table S1. Physicochemical properties of olive oil soft soap formulated with varying levels of *Lavandula dentata* essential oil

Samples	Moisture content %	pH	Free alkali content test %	Alcohol insoluble matter %
**Soap**	25.86 ±1.3 [a]	10.04 ± 0.03 [a]	0.70 ± 0.03 [b]	1.84 ± 0.026 [a]
**Soap with 0 % extra virgin olive**				
**5% *L. dentata* EO**	24.78 ± 0.7[a]	10.68 ± 0.04 [a]	0.061 ± 0.008 [a]	1.76 ± 0.019 [a]
**8% *L. dentata* EO**	25.15 ± 1.4[a]	10.14 ± 0.07 [a]	0.048 ± 0.003 [a]	1.73 ± 0.028 [a]
**12% *L. dentata* EO**	25.15 ± 1.9 [a]	10.61 ± 0.02 [a]	0.045 ± 0.003 [a]	1.56 ± 0.063 [a]
**Soap with 5 % extra virgin olive**				
**5% *L. dentata* EO**	25.32 ± 1.3 [a]	10.75 ± 0.02 [a]	0.076 ± 0.008 [a]	1.66 ± 0.014 [a]
**8% *L. dentata* EO**	24.89 ± 1.32 [a]	10.09 ± 0.07 [a]	0.046 ± 0.002 [a]	1.63 ± 0.082 [a]
**12% *L. dentata* EO**	25.94 ± 1.2 [a]	10.03 ± 0.06 [a]	0.089 ± 0.003 [a]	1.83 ± 0.022 [a]

Data are expressed as mean values ± standard deviation. a–b: means in the same column with different letters are significantly different ($P < 0.05$).

Table S2. Antioxidant activity (DPPH assay) of *Lavandula dentata* essential oil, extra virgin olive oil, BHT, and formulated soap samples

Sample	DPPH IC_{50} (mg/mL)	DPPH inhibition (%)
BHT (positive control)	0.0146 ± 0.0008 [a]	—
L. dentata EO	8.72 ± 0.03 [b]	—
Extra virgin olive oil (EVOO)	9.25 ± 0.19 [c]	—
Soap, 0% EVOO – 0% EO	—	9.09 ± 3.27 [a]
Soap, 0% EVOO – 5% EO	—	12.48 ± 2.64 [b]
Soap, 0% EVOO – 8% EO	—	17.75 ± 1.03 [c]
Soap, 0% EVOO – 12% EO	—	20.40 ± 1.09 [d]
Soap, 5% EVOO – 0% EO	—	14.05 ± 0.15 [a]
Soap, 5% EVOO – 5% EO	—	15.83 ± 0.52 [b]
Soap, 5% EVOO – 8% EO	—	18.71 ± 0.19 [c]
Soap, 5% EVOO – 12% EO	—	26.67 ± 0.32 [d]

Values are expressed as mean ± SD ($n = 3$). Different superscript letters indicate statistically significant differences within each group of comparable samples (one-way ANOVA followed by Tukey's post-hoc test, $p < 0.05$). Lower IC_{50} values and higher inhibition percentages indicate greater radical-scavenging efficacy.
